# Critical points of DNA quantification by real-time PCR – effects of DNA extraction method and sample matrix on quantification of genetically modified organisms

**DOI:** 10.1186/1472-6750-6-37

**Published:** 2006-08-14

**Authors:** Katarina Cankar, Dejan Štebih, Tanja Dreo, Jana Žel, Kristina Gruden

**Affiliations:** 1 Department of Plant Physiology and Biotechnology, National Institute of Biology, Večna pot 111, 1000 Ljubljana, Slovenia; 2 Department of Biochemistry and Molecular Biology, Jožef Štefan Institute, Jamova 39, 1000 Ljubljana, Slovenia

## Abstract

**Background:**

Real-time PCR is the technique of choice for nucleic acid quantification. In the field of detection of genetically modified organisms (GMOs) quantification of biotech products may be required to fulfil legislative requirements. However, successful quantification depends crucially on the quality of the sample DNA analyzed. Methods for GMO detection are generally validated on certified reference materials that are in the form of powdered grain material, while detection in routine laboratories must be performed on a wide variety of sample matrixes. Due to food processing, the DNA in sample matrixes can be present in low amounts and also degraded. In addition, molecules of plant origin or from other sources that affect PCR amplification of samples will influence the reliability of the quantification. Further, the wide variety of sample matrixes presents a challenge for detection laboratories. The extraction method must ensure high yield and quality of the DNA obtained and must be carefully selected, since even components of DNA extraction solutions can influence PCR reactions. GMO quantification is based on a standard curve, therefore similarity of PCR efficiency for the sample and standard reference material is a prerequisite for exact quantification. Little information on the performance of real-time PCR on samples of different matrixes is available.

**Results:**

Five commonly used DNA extraction techniques were compared and their suitability for quantitative analysis was assessed. The effect of sample matrix on nucleic acid quantification was assessed by comparing 4 maize and 4 soybean matrixes. In addition 205 maize and soybean samples from routine analysis were analyzed for PCR efficiency to assess variability of PCR performance within each sample matrix. Together with the amount of DNA needed for reliable quantification, PCR efficiency is the crucial parameter determining the reliability of quantitative results, therefore it was chosen as the primary criterion by which to evaluate the quality and performance on different matrixes and extraction techniques. The effect of PCR efficiency on the resulting GMO content is demonstrated.

**Conclusion:**

The crucial influence of extraction technique and sample matrix properties on the results of GMO quantification is demonstrated. Appropriate extraction techniques for each matrix need to be determined to achieve accurate DNA quantification. Nevertheless, as it is shown that in the area of food and feed testing matrix with certain specificities is impossible to define strict quality controls need to be introduced to monitor PCR. The results of our study are also applicable to other fields of quantitative testing by real-time PCR.

## Background

In a decade of use of genetically modified organisms (GMOs) the global area of biotech crops increased to 90 million hectares in 2005, and the area sown continues to increase [1]. Due to low public acceptance of GMOs in many countries, several governments have implemented, or are in the process of adopting, legislation that requires traceability of GM components and labelling of products that contain GMOs above a certain threshold. Therefore quantitative techniques had to be developed and implemented.

The method of choice for gene quantification is real-time PCR. This technique proves to be more suitable for the diagnostic laboratory than conventional PCR, due to its quantitative performance, greater sensitivity and the use of closed-tube assays. Its use in quantitative analysis of genetically modified organisms has been reviewed [2-4]. The most frequent quantitative application of real-time PCR is in gene expression studies. In this case the interest is in the order of changes in expression, and the limit of detection has been reported as being a two fold difference [5]. However, greater accuracy is required in GMO diagnostics, therefore it is necessary to be fully aware of the factors influencing quantification since inaccurate analyses can result in liability issues. The choice of DNA extraction procedure can crucially influence the quantifiability of GMOs, but the choice of extraction method is often a trade-off between costs, optimal yield of DNA and removal of substances that could influence the PCR reaction. A procedure that results in an optimal yield of DNA and in removal of substances that could influence the PCR reaction is essential. DNA extraction techniques have been compared [6-8]. Methods for GMO analysis are normally validated on only one sample type, typically powdered grain material, or on a very limited range of sample matrixes [4,9]. In reality, GMO laboratories are faced with a broad spectrum of different foodstuffs, from raw plant materials to highly processed food products.

A new, modular approach to method validation has been proposed by Holst-Jensen and Berdal [9] in which each step of the analytical procedure is validated as a separate module. According to this approach validation of the extraction procedure for different sample matrixes is necessary and is done by assessing the ability of the extraction method to provide suitable DNA from the specific matrix. The modular approach would also lead to validation of the extraction procedure for different sample matrixes by assessing the ability of the extraction method to provide suitable DNA from each. Many new extraction techniques appropriate for a certain matrix have been proposed [10-12] and the matrix effect discussed [4,9]. Nevertheless, a matrix in GMO analysis is very hard to define since the same product (e.g. tortilla chips or bread) produced by two different procedures can have a different composition and contain different substances that could affect efficiency of PCR. Only rarely does the laboratory receive complete compositional data on the products, so the choice of extraction technique is usually based on previous experience with similar samples. Reliable controls must therefore be included for each sample to ensure reliability of results.

The DNA extracted from different sample matrixes must be evaluated for suitability in quantitative analysis. The first criterion is that sufficient DNA must be available to guarantee reliable detection of the transgene at the threshold legislated in different countries [13,14]. The second criterion concerns the quality of the extracted DNA. Substances present in plant and food samples that can be co-extracted with DNA and can affect PCR include polysaccharides, proteins, phenolic compounds and other plant secondary metabolites [7,15-17]. Additionally, components of DNA isolation buffers can affect PCR reactions [16]. The effect of co-extracted substance can result in inhibition of the PCR reaction, where amplification is blocked completely or the Ct values obtained in the PCR reaction are higher then expected from the number of target molecules added to the reaction, or a decrease of overall efficiency of PCR reaction is observed [18]. But the effect can also be demonstrated as facilitation of PCR amplification [15]. The quality of DNA concerns not only the purity but also the structural integrity of DNA obtained and the term PCR forming units (PFU) was introduced to distinguish between structurally intact and damaged (non-amplifiable) target DNA copies [9].

Two general approaches for the quantification of nucleic acids, the comparative Ct approach and the standard curve approach [19], are currently in use in GMO detection. In the first, a validation experiment must first be performed that demonstrates that the efficiency of target gene and the reference gene are approximately equal. A linear relationship is established on the basis of the difference in Ct value of the reference gene and the GMO target, respectively, using e.g. certified reference materials (CRMs) covering a range of defined concentrations of the GMO material. The assumption inherent in this method is that the amplification efficiencies of the reference gene and the GM amplicon are the same in all subsequent experiments for all samples analysed. The approach therefore needs to be very well validated and can be used only on the raw material or on a limited number of matrixes in which the variability in the terms of quality of obtained DNA presumably does not vary. Since GMO laboratories are faced daily with different products this approach is, in most cases, inappropriate. In the so called "standard curve" approach standard curves for the quantification of the reference gene and the GM target are prepared separately using serial dilutions of one standard reference material DNA. In this way the difference in PCR efficiency between the amplicons is evaluated. This approach is more robust, since the amplification efficiency of different amplicons is taken into account. However the efficiency of the PCR reaction is still presumed to be the same for the standard reference material and for the sample analyzed. Differences in the PCR efficiencies between the reference material and the sample lead to under- or over-estimation of GMO content.

The modular approach was used in our study to better determine extend of individual parameters that can influence the quantification of nucleic acids by real-time PCR. Our hypothesis was that the reliability of the quantitative analysis may depend crucially on the properties of the sample itself, on efficient homogenization of the sample, the DNA extraction procedure and finally on the performance of the real-time PCR reaction. The purpose of our study was to analyse the effect of some of these parameters on GMO quantification. Five DNA isolation protocols were compared with respect to the influence of the extraction procedure on real-time PCR efficiency and, consequently, the GMO quantification. The effect of the sample matrix on PCR efficiency was studied on different soybean and maize matrixes. Additionally the first assessment of variability of PCR efficiency within sample matrixes is presented. PCR efficiency was considered as the primary criterion in assessing the quality of extracted DNA. The influence of variable PCR efficiency on final GMO percent estimate is demonstrated.

## Results and discussion

### Influence of intra- and inter-run variability of real-time PCR reactions on quantification of GMO

To enable the effect of different sample matrixes and different extraction procedures on quantification of GMOs to be studied, the intra-run and inter-run variability of the real-time PCR reactions was first determined. Quantity of taxon specific gene and the transgene was determined on 5% certified reference material of GTS 40-3-2 (Roundup Ready^® ^soybean; RRS) soybean at different DNA concentration levels. Standard deviations (SD) and coefficients of variation (CV) were calculated, first within one real-time PCR run. A typical experiment is shown in the left part of Table 1. The efficiency of amplification was 0.92 for the RRS amplicon and 0.97 for the lectin amplicon. Low CVs of copy number estimates were obtained in all experiments with a gradual increase of variability up to CVs above 15% at high low genome copy numbers per reaction.

Inter-assay variability was calculated from 10 independent real-time PCR runs (Table 1). The average slope of the standard curves was -3.43 ± 0.08 for the lectin amplicon and -3.51 ± 0.16 for the RRS amplicon indicating efficiencies of 0.96 ± 0.03 and 0.92 ± 0.05, respectively. Coefficients of variation of copy number estimates for ten independent real-time runs were below 15%, except when only few molecules of the target are added to the reaction and results are influenced by a stochastic effect. These measurements are considered to be outside the range of quantification of the method, which was determined to be 30 copies of the target for RRS amplicon.

The content of GMOs is calculated by dividing the amount of transgene by the amount of the species specific gene (e.g. the amount of RRS specific amplicons with the amount of lectin gene specific for soybean) therefore the CVs of both amplicons affect the final result. The estimated CV attributed to the final result ranged from 4.4% to 12.5%. In samples with lower GM content the number of transgene copies added to one reaction is lower therefore higher CV is expected. All calculations (also in subsequent chapters) are based on copy number estimates so it must be noted that in cases where DNA quantity per soybean mass unit differs between the GM and non-GM material in the sample, the DNA based GM quantity will deviate from the quantity determined by mass to mass ratio of GM and non-GM material [20].

### Effect of the DNA extraction method on PCR efficiency

We compared 5 different extraction techniques (see methods section) commonly used in GMO detection laboratories. Certified reference material of RRS was chosen for this comparison, since it is an unprocessed sample with defined particle size. The effect of extraction procedure on PCR efficiency, and consequently on GMO quantification, was evaluated. 4 different target sequences were amplified from extracted DNA (lectin, p35S, NOS and RRS specific target).

**Figure 1:**
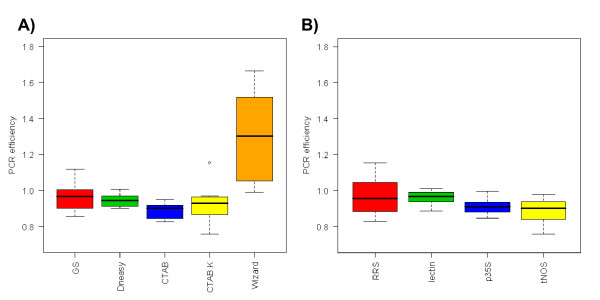
**Influence of the DNA extraction method on PCR efficiency**. (A) Variability of PCR efficiency for different DNA isolation methods. Outlier for the CTAB procedure with proteinase K and RNase A treatment is shown as circle above the boxplot. (GS = GENESpin, CTAB.K = CTAB procedure with proteinase K and RNase A treatment). (B) The distribution of PCR efficiencies of 4 tested amplicons on different DNA extracts is presented in boxplots (efficiency data for DNA isolated with Wizard method was excluded because of high variability of results).

The PCR efficiency for different DNA extracts was first compared (Figure 1A). For most extraction procedures the efficiencies were close to 1 for all four tested amplicons, indicating that these extraction methods are suitable for quantification. A distinct dispersion of efficiency values and decline from optimal efficiency was observed for the Wizard extraction. Although this extraction provided amplifiable DNA, the high fluctuation in PCR efficiency could lead to over- or under-estimation of GMO content. The dispersion of efficiency values was also compared for different amplicons (Figure 1B). The results obtained for the Wizard extraction method have been removed from evaluation of amplicons due to high dispersion of the data. CVs attributed to individual amplicons on the basis of remaining data were 1%, 4%, 5% and 10% for lectin, p35S, tNOS and RRS, respectively, indicating the same level of robustness for all four amplicons if a proper DNA extraction procedure used.

### Effect of sample matrix on PCR efficiency

GMO detection methods are usually validated using standard reference materials or plant material. GMOs on the other hand have to be detected in a variety of sample matrixes. To assess the effect of the sample matrix on GMO quantification we analyzed DNA in four maize and four soybean samples. Two unprocessed samples were chosen: flour (CRM) and grains. Tortilla chips and soybean milk were chosen as processed samples of maize and soybean. In addition, an example of feed sample containing either soybean or maize was analyzed. The samples were compared to monitor differences in PCR efficiency and to detect possible effect on PCR amplification. PCR efficiency was calculated from a regression line of Ct values obtained from dilutions of each matrix DNA within the previously determined dynamic range of the method (Table 2). Higher Ct values were obtained for food matrixes compared to CRM material due to lower amount of DNA isolated from these samples. Lower correlation coefficient of regression lines were observed for RRS amplicon due to lower target copy number per reaction.

**Figure 2:**
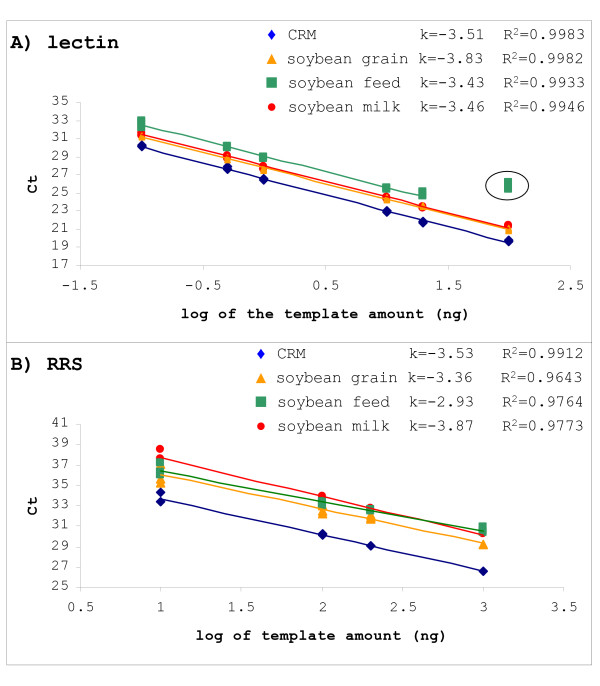
**Efficiency of amplification in 4 soybean matrixes**. Standard curves made by serial dilution of DNA isolated from 4 sample matrixes are shown for (A) the species specific gene (lectin) and (B) the transgene (RRS). The position of RRS curves for food materials is shifted from the CRM standard curve due to lower content of GM material in spiked food samples, as expected. The slope and correlation coefficient of the linear regression line are given. The highest concentration of soybean feed DNA was inhibited in the lectin and therefore excluded from the standard curve (encircled in plot A).

For maize, all matrixes showed a PCR efficiency that was close to that of the certified reference material, for both endogenous gene amplicon (invertase) and GMO specific amplicon. However, in the case of soybean matrixes, inhibition was detected for soybean feed sample (Figure 2). The highest concentration of soybean feed DNA was inhibited in the case of the endogenous gene (lectin) and therefore had to be excluded from the regression line, while the PCR efficiency of the RRS amplicon for this sample showed a substantially higher PCR efficiency than that of the certified reference material. Although the DNeasy isolation method used in this case normally provides high quality DNA from feed matrixes, in this case the results would be biased and would lead to overestimation of the RRS content. In the case of soybean grain we did not expect a reduced PCR efficiency since the matrix is unprocessed, however the lowest efficiency was obtained for this matrix with the lectin amplicon. A possible cause for reduced performance of PCR on grain samples could be the inability of used extraction methods to compensate for the high lipid content in the sample of soybean grain. Similar effects have also often been observed in routine analysis when grains are treated with chemicals such as fungicides.

**Figure 3:**
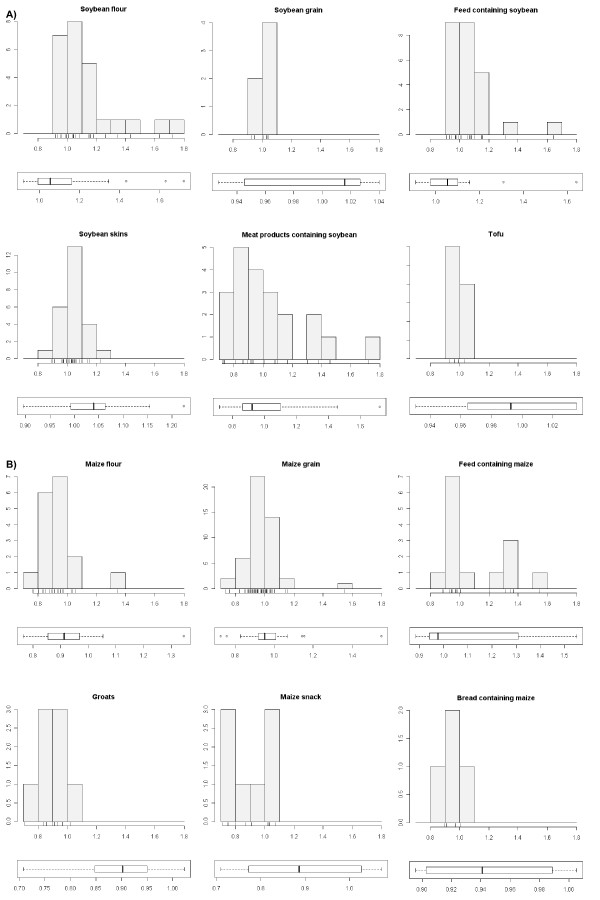
**Variability of PCR efficiency within matrixes**. PCR efficiencies for soybean (A) and maize (B) matrixes were determined for the plant specific genes, lectin and invertase, respectively. The dispersions of PCR efficiencies is shown in a histogram with PCR efficiency on the x axis and number of samples on the y axis. For a scaled view and detection of outliers, boxplots of efficiency data for each matrix are presented below each histogram.

To evaluate the influence of the matrix on a larger scale, 205 samples from routine analyses, representing 6 maize and 6 soybean matrixes, were assayed for PCR efficiency by preparing sample dilutions (Figure 3). Although the majority of the samples show a PCR efficiency close to 1 for each of the sample matrixes, a substantial variability is evident from the plots. Although the extraction method can be considered as validated for a certain matrix, DNA of different samples is not necessarily equally suitable for quantitative analysis.

The above results suggest that each extraction method should be validated for different matrixes. The range of matrixes for which an extraction protocol can be applied should be defined. The definition of a matrix remains elusive and even a simple matrix, as for example grain, can vary greatly in content of moisture, fibre, starch or chemical residues [4]. For processed food matrixes the situation is even more complex since the same food matrix (e.g. tortilla chips or soybean milk) can vary greatly in composition from one food producer to another. The degradation and fragmentation of the DNA in the matrix can differ, depending on the processing procedure and consequently limit the amount of DNA that can be extracted from the sample. Low amount of DNA obtained can increase the measurement uncertainty of GMO estimation due to high variability of quantity estimations caused by occurrence of stochastic effects, therefore the quantities should always be measured within the dynamic range of the method where the calculated CV should not exceed a certain acceptance value (e.g. 25%). The effect on PCR amplification can also be observable over-amplification, i.e. amplification efficiency > 1, which could also be explained by a compound or structural conformation that leaves template copies inaccessible for the DNA polymerase in the initial PCR cycles and gradual conformation changes make more and more template copies accessible.

This leads us to consider the definition of a matrix, since only a food product that has been produced by an unchanged procedure with defined contents can be properly validated. Such definition is only suitable for implementing product control in the factories but, even there, changes occur in the production of food products due to lot to lot differences in food constituents. From the perspective of a detection laboratory such fragmentation in defining matrixes and identifying substances that affect PCR in each product is impossible. DNA extraction methods can be defined that are most suitable for removal of potential substances that could affect PCR amplification like polysaccharides, lipids or phenolic compounds and are suitable for those sample matrixes that contain them. Additionally appropriate controls of extracted DNA PCR efficiency should be introduced with each analysis.

### Differential matrix influence on PCR efficiency of amplicons

**Figure 4:**
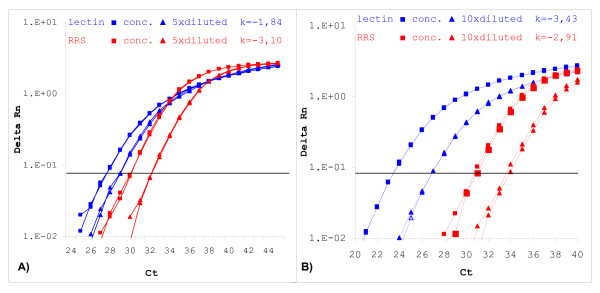
**Differential effect on PCR efficiency of amplicons**. The differential influence on PCR reaction for two amplicons is shown for two samples of soybean obtained in routine analyses. Although the same amount of DNA was added to each PCR reaction, inhibition was evident for the lectin amplicon but not for the RRS specific amplicon for a soybean feed sample (A). In the case of soybean flour (B), efficiency of the PCR reaction was lower for the RRS amplicon and differed substantially from that for the lectin amplicon. (k = calculated slope of the standard curve linear regression line).

It is generally assumed that changes in PCR efficiency occur due to substances in the isolated DNA, such as enhancers and inhibitors of the PCR reaction, originating either from the sample matrix or from the DNA extraction solutions. Similar changes in PCR efficiency should be exhibited for all amplicons tested when the same amount of extracted DNA is added to the reaction. However, differential effect on one amplicon can sometimes be observed, as shown in Figure 4. Two routine samples are presented showing opposite effects. In the case of soybean feed sample the lectin amplicon was affected but not the RRS amplicon, while in the case of the soybean flour sample, the RRS amplicon was affected more severely. The amount of DNA extract added to the reaction was the same for both amplicons and thus also the amount of substances that affect the PCR. All measurements are within the dynamic range of quantification determined for these two amplicons. The difference can also not be explained by interference with the fluorescence detection step of the reporter dye, as the reporter and quencher dyes used are the same for both amplicons.

Recommendations for quantitative PCR [21] suggest a preliminary real-time PCR run for each sample to be quantified, by assaying dilutions of the sample for the endogenous reference gene to detect PCR effectors. The so-called "monitor run" does not however take account of differential change in efficiency for different amplicons, so additional controls should be included to check amplification efficiency for each individual amplicon used for quantitative analysis.

### Influence of real-time PCR efficiency on the final GMO percent estimation

In our evaluation of extraction and matrix effects on GMO quantification, PCR efficiency was identified as the primary parameter that determines the suitability of the DNA for quantitative analysis. In Table 3 the effect is demonstrated on a theoretical model case of an RRS sample. In the case of optimal (1.0) PCR efficiency of the standard curve we have simulated variability by varying the efficiency for the species specific and GM specific amplicons, and observed the consequent changes in the resulting GMO content. The GMO content can clearly only be determined correctly when the PCR efficiency of the sample is the same as for the standard curve. In a case where the efficiency for the sample differs from that for the standard curve, the GMO estimates diverge increasingly from the true value, with higher differences in efficiency. Even greater differences are obtained when the amplicons are affected differently, as shown in the last column of Table 3, where only the efficiency of the transgene was varied.

**Figure 5:**
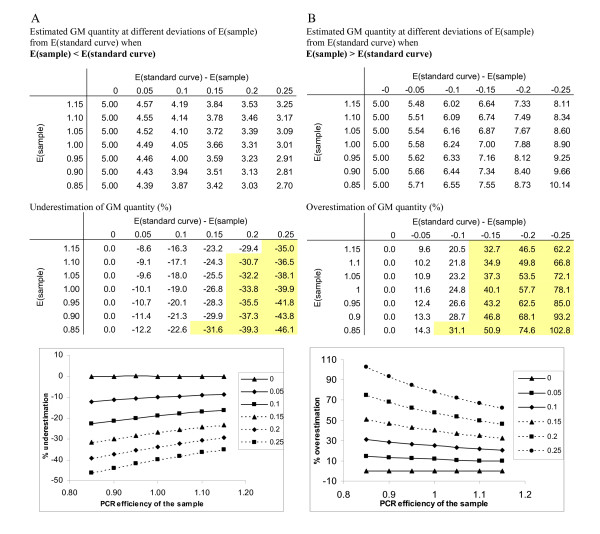
**Influence of PCR efficiency on accuracy of GM content determination**. The effect of PCR efficiency on the estimated GM content is shown for a theoretical case of 5% GMO when (A) PCR efficiency of the sample is higher then the PCR efficiency of the standard curve and when (B) the PCR efficiency of the sample is lower then the PCR efficiency of the standard curve. Effect of the difference of PCR efficiencies was modelled when E(sample) deviates form E(standard curve) for 0, 0.05, 0.10, 0.15, 0.20 and 0.25. Upper table (in both A and B) shows the determined GM percentage at different deviations of PCR efficiency. Lower table shows the deviation of the estimated GM content from true value in %. Results that deviate from true value for more then 30% are shaded in yellow. In the plots dependency of the of under- or overestimation of the GM content on the PCR efficiency of the sample is shown when E(sample) deviates form E(standard curve) for 0, 0.05, 0.10, 0.15, 0.20 and 0.25. (E = PCR efficiency).

Additionally, assumption that the final result is not affected when both targets are affected to the same extend (e.g. the efficiency for both lectin amplicon and RRS amplicon in the sample is reduced from 1 to 0.90 compared to the standard curve) was tested. A theoretical case of quantification of 5% RRS was considered and two situations were modelled: a case where the E(sample) (PCR efficiency of the sample) is higher then the E(standard curve) (PCR efficiency of the standard curve) and a case when the E(sample) is lower then the E(standard curve). The effect on the determined GM quantity was determined at differences between the E(sample) and E(standard curve) of 0, 0.05, 0.10, 0.15, 0.20 and 0.25 for both amplicons. Results are shown in Figure 5. The estimated GM percent diverges from true value with increasing difference between the E(sample) and E(standard curve), although this difference is the same for both amplicons. The deviation from the true value is higher at low PCR efficiencies. 30% accuracy level can be reached when E(standard curve)-E(sample) is between -0.1 and 0.15 at 5% GM content.

**Figure 6:**
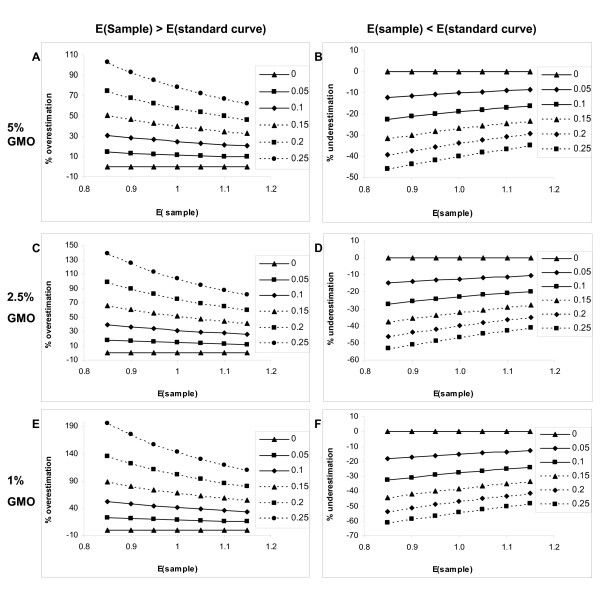
**Influence of PCR efficiency on GM content determination at different GM concentration ranges**. Deviation of the estimated GM content from the true value (in %) is shown when E(sample) deviates form E(standard curve) for 0, 0.05, 0.10, 0.15, 0.20 and 0.25. Overestimation of GM content (in %) when PCR efficiency of the sample E(sample) is higher then the PCR efficiency of the standard curve is shown for 5% (A), 2.5% (C) and 1% GMO (E). Underestimation of GM content (in %) when PCR efficiency of the sample E(sample) is lower then the PCR efficiency of the standard curve is shown for 5% (B), 2.5% (D) and 1% GMO (F). (E = PCR efficiency).

The accuracy of relative quantity estimates also depends on the ratio between the species specific gene and the transgene. We modelled the deviation from the true value for 5%, 2.5% and 1% GMO levels (Figure 6). As the ratio between the species specific gene and the transgene decreases, higher similarity of E(sample) to E(standard curve) is required to reach the same level of accuracy. For 30% accuracy E(standard curve)-E(sample) must be between -0.05 and 0.1 at 2,5% GM level and between -0.05 and -0.05 at 1% GM level. The model provides an objective way to define acceptable amplification efficiency ranges according to the accuracy level desired. The effect can although be compensated if in such cases higher dilutions of DNA are added in reference gene quantification. This example demonstrates that it is essential to measure PCR efficiency for each sample if exact quantification is to be achieved. Detailed study of the matrix and DNA isolation effects will also contribute to better estimates of the measurement uncertainty associated with these factors.

## Conclusion

In this paper some critical points in GMO quantification are highlighted and their influence on the final results demonstrated. Real-time PCR is the most precise method available for gene quantification. In addition to the intra-run and inter-run variability which is relatively low of the real-time PCR reaction however, its performance is substantially influenced by the sample properties. DNA of high quality is required for accurate quantification.

First of all, the DNA isolation procedure can substantially influence the quantification, since different methods differ in their effectiveness in removing substances that interfere with the PCR reaction. In addition, components of the DNA isolation solutions can themselves influence PCR reactions. Therefore the appropriate choice of an extraction method suitable for a particular sample matrix is a prerequisite for successful downstream analysis. As a sample matrixes are highly variable and difficult to define exactly, optimisation of DNA isolation procedures for each matrix is impossible. Additionally, we have shown that the efficiency of amplification is not necessarily influenced in the same way for all amplicons. These can lead to even larger errors in quantification. We conclude that appropriate controls must be included in PCR quantification to evaluate the suitability of the isolated DNA for quantitative analysis. GMOs are normally quantified by using a standard curve produced by dilutions of standard reference materials but the efficiency of amplification for the sample is rarely assessed. For exact quantification these efficiencies should be the same. Our results suggest this is rarely so and that a lot of variation is present within individual matrixes. Dilution controls should therefore be included for each sample quantified, preferably for each amplicon being assayed, due to occurrence of differential effect on efficiency of different amplicons. The inclusion of such controls poses two problems. Firstly, objective acceptance criteria are needed to compare the efficiency of the sample to that of the standard curve, in a manner similar to that already proposed for the comparison of standard curves [22]. This would determine whether the quantification is feasible. The second problem is the additional cost of such an analysis, especially in the case of plant species where a lot of GMO lines are on the market. We recommend using such approach when exact quantitative results are needed even though it is associated with additional cost of analysis. Other simplifications like multiplexing or eliminating samples that clearly exceed or are below labelling threshold should be implemented to reduce costs instead.

This may require the introduction, for routine analysis, of methods that determine PCR efficiency from the real-time PCR response curves in single reaction set-up [23-25]. The idea is attractive for routine real-time PCR analysis since reliable corrections for PCR efficiency can be implemented for each separate PCR reaction and standard curves and sample dilutions could be omitted which would substantially reduce the cost of analysis. Due to advances in this area determination of PCR efficiency from the amplification curves may become reality sometime in the near future [18].

The data obtained in our experiments provide experimental evidence for the positive value of the modular approach to validation studies and show an example on how the DNA extraction module and PCR amplification module can be independently assessed.

## Methods

### Genetically modified plants and food samples

Powdered certified reference materials (CRMs) for genetically modified maize (*Zea mays*) event MON810 and GTS 40-3-2 soybean (*Glycine max*) were purchased from the Institute for Reference Materials and Measurements (IRMM, Geel, Belgium). Samples included grains of soybean and maize and animal feed containing either soybean or maize, and processed food samples of soybean milk and tortilla chips as examples of food and feed matrixes. Food and feed samples were purchased in local stores. Data obtained from routine GMO detection of 98 maize samples, with differing sample matrixes, was assessed statistically: grains (47 samples), groats (8), flour (17), animal feed (14), maize snack (8), bread (4) and 107 samples that contain soybean: grains (6), flour (25), soybean skins (25), animal feed (25), tofu(5), and meat products containing soybean flour or soybean proteins (21).

### DNA isolation and quantification

DNA was isolated from 5% RRS CRM, following five protocols: DNeasy Plant Mini kit (Qiagen, Valencia, CA), Wizard extraction [26] (Promega, Madison, WI), CTAB based extraction [27], modified CTAB extraction (see below) and GENESpin kit (GeneScan, Freiburg, Germany). 100 mg samples were used for all isolations, except for the GENESpin isolation where 200 mg was used, as recommended in the producer's manual.

DNA isolation with DNeasy Plant Mini Kit was performed according to the producer's manual with the incubation time of the sample in lysis buffer extended to 20 minutes. Two CTAB procedures were compared, one following the standard protocol [27], and one omitting proteinase K and RNase A treatment. All DNA extractions were performed in duplicate.

DNA of 5% CRM for RRS used for determination of the inter- and intra-run variability was isolated with GENESpin kit. DNA was isolated from samples of CRM, grain, feed, tortilla chips and soybean milk using DNeasy Plant Mini Kit (Qiagen, Valencia, CA), since this method was previously successfully applied for isolation of DNA of all assayed matrixes in routine analysis. All matrixes but CRMs used were confirmed to be GM negative by screening for the presence of p35S. Samples of grains, feed and processed food were spiked with 160 ng of DNA extracted from 5% RRS CRM or with 310 ng of DNA of 5% MON810 CRM. In both cases spiked material constituted 10% of the final volume of DNA extract.

DNA from food samples obtained from routine testing was isolated with GENESpin kit. DNA was quantified using Picogreen fluorescent dye (Molecular Probes Inc., Eugene, OR) by measuring fluorescence at 535 nm in a Tecan fluorimeter (Tecan, Crailsheim, Germany).

### Quantitative real-time PCR

Lectin [28] and invertase [29] genes were used as endogenous control genes for soybean and maize respectively. Primers and probe specific for p35S promoter [28,30] and tNOS terminator [31] were used to target common promoter and terminator sequences. Primers and probe construct specific for RRS [28] and event specific for genetically modified maize MON810 [32] were used. Sequences of primers and probes used are listed in Table 4.

Taqman^® ^probes (Applied Biosystems, Foster City, CA) were labelled with 5'-FAM and 3'-TAMRA, with the exception of the invertase probe that was labelled with 5'-VIC. A 20 μl PCR reaction containing 2× TaqMan^® ^Universal MasterMix (Applied Biosystems, Foster City, CA) was performed on an AbiPrism7900 Instrument. Following assay optimization, primer and probe concentrations used were: 900 nM primer and 200 nM probe for amplification of lectin and invertase genes and 600 nM primer and 150 nM probe for p35S, tNOS, RRS and MON810 amplicons. Reactions were performed under standard conditions [33]. Results were analyzed using SDS 2.1 software (Applied Biosystems, Foster City, CA) after manual adjustment of the baseline and fluorescence threshold.

### Assessment of intra- and inter-run real-time PCR variability

Inter- and intra-run variability assessment was performed on 5% RRS standard reference material, diluted to contain approximately 100, 20, 5, 1 and 0.5 ng of DNA which corresponds to approximately 87000, 17400, 4350, 870 and 435 soybean genome copies per reaction on the basis of the soybean genome size (1C) [34]. Intra-run variability was assessed by running real-time PCR reactions in triplicate for each dilution, targeting lectin and RRS amplicons. Standard deviations and coefficients of variation were calculated for the Ct values and for the estimated copy number. Inter-run variability was assessed by repeating the assay in 10 independent real-time PCR runs. Identical fluorescence threshold and baseline settings were used for comparability of results. Ct and estimated copy number were averaged over the 10 runs for each of the dilutions. Intra-run variability of Ct values and estimated copy numbers were evaluated from the standard deviation and coefficient of variation. Efficiency of amplification was calculated for each real-time PCR run of each amplicon, as described below. Copy number was calculated by interpolation of generated Ct values generated in a standard regression curve.

### Effect of DNA extraction method on quantitative real-time PCR

DNA from two replicate extracts of 5% RRS CRM obtained with each of the five extraction methods tested was dilute to contain approximately 100, 20, 4, 0.8 and 0.16 ng of DNA, corresponding to 87000, 17400, 3480, 790, and 140 copies of soybean genome on the basis of the soybean genome size (1C) [34]. Dilutions were assayed by real-time PCR using primers and probes specific for lectin, p35S, tNOS and RRS construct specific amplicons. All PCR reactions were run in triplicate. Ct values were plotted against log_10 _of amplicon amount and amplification efficiencies were calculated from the slopes of the standard curves.

Results are shown in the form of a boxplot, where the box contains the middle 50% of the data. The upper edge (hinge) of the box indicates the 75th percentile of the data set, and the lower hinge indicates the 25th percentile. The ends of the vertical lines or "whiskers" indicate the minimum and maximum data values, unless outliers are present in which case the whiskers extend to a maximum of 1.5 times the inter-quartile range.

### Effect of sample matrixes on quantitative real-time PCR

DNA from soybean and maize samples was diluted to contain approximately 100, 20, 10, 1, 0.5 and 0.1 ng of DNA. All reactions were run in triplicate. Using quantitative real-time PCR, soybean samples were assayed using lectin amplicon and RRS amplicon and maize samples assayed for the presence of invertase and MON810 maize. Two highest dilutions of the sample were below the quantification limit for RRS and MON810 amplicons and were therefore excluded from regression lines for these amplicons. Data from routine samples were assessed for variation in PCR efficiency. Efficiency of the real-time PCR reaction was calculated as described below using Ct values obtained by 10× dilutions of the sample DNA. The efficiency was evaluated based on the standard reference gene (lectin or invertase).

### Influence of real-time PCR efficiency on the estimation of GMO content

The influence of PCR efficiency on GMO quantification was assessed on a theoretical case by performing the following calculation. 100 ng of 5% heterozygous RRS DNA, containing 87000 copies of soybean specific gene and 4350 copies of the transgene, were assumed to be added to a reaction. The sample was compared to a standard curve obtained with 100% amplification efficiency for both the soybean specific amplicon and for the transgene. Fluorescence measurements for 100 ng of soybean DNA are known, from experience, to cross the threshold at approximately cycle 22 for lectin gene. In this cycle approximately 3.6 × 10^11 ^molecules of the amplicon are formed, so this number was taken as the detection threshold for our calculation. The number of cycles needed to reach the threshold was calculated at different efficiencies for both amplicons tested. The initial copy number estimate for the RRS and lectin amplicons was then calculated and the amount of GMO material estimated by dividing the number of copies of the transgene by the number of copies of the species specific gene.

Further we theoretically assessed the effect of efficiency changes on GMO quantification at PCR on the case of 5%, 2.5% and 1% GMO. The efficiencies (E) of the sample were ranging from 0.85 to 0.15 and the difference between the E(sample) and E(standard curve) of 0, 0.05, 0.10, 0.15, 0.20 for both species specific and transgene specific amplicons. Situations where E(sample) is higher and lower then E(standard curve) were modelled. The initial copy number estimate for the transgene and species specific amplicons was calculated and the amount of GMO material estimated by dividing the number of copies of the transgene by the number of copies of the species specific gene, as described above. Deviation from true value (%) was calculated and plotted against the PCR efficiency of the sample.

### Efficiency calculation and data analysis

Standard curves were constructed by plotting Ct values against log_10 _of DNA amount and fitted by linear least square regression. Individual measurements for replicate real-time PCR reactions were plotted separately as suggested by Burns et al. [35] to obtain correlation coefficients (R^2^) that reflect the variation of the data set. The slope of the linear regression line was used to calculate real-time PCR efficiency from E = 10^[-1/slope] ^- 1 [36,37], where a PCR efficiency of 1 indicates the highest efficiency, where all target molecules double in one PCR cycle, and an efficiency of 0 indicates no amplification. Standard deviations and coefficients of variation were evaluated for replicate measurements. The coefficient of variation that would be attributed to the final GMO percent calculation was estimated from: CV = √(CV_reference gene_^2 ^+ CV_target gene_^2^) [19]. Data were analyzed by basic spreadsheet software and R statistical software [38].

## Abbreviations

CRM: certified reference materials

Ct: threshold cycle

CV: coefficient of variation

E: PCR efficiency

FAM: 6-carboxyfluoresceine

GM: genetically modified

GMO: genetically modified organism

PCR: polymerase chain reaction

RRS: Roundup Ready^® ^soybean line GT 40-3-2

SD: standard deviation

TAMRA: 5-carboxytetramethylrhodamine

## Authors' contributions

KC conducted laboratory experiments, analyzed the data and drafted the manuscript. TD helped with experimental work in the laboratory and participated in design of the experiments. DŠ gathered and contributed data about PCR efficiency from routine GMO analysis. JŽ participated in the design of the study and helped in drafting the manuscript. KG participated in the design of the study, data analysis and drafting the manuscript. All authors read and approved the final manuscript.

## Figures and Tables

**Table 1:** Intra- and inter-run variability of real-time PCR reactions. Intra- and inter-run variability was assayed for 5% RRS standard reference material, diluted to contain approximately 100, 20, 5, 1 and 0.5 ng of DNA. (Ct = Cycle threshold, SD = standard deviation of estimated copy number, CV = coefficient of variation of estimated copy number [%],* measurements outside the quantification range of the method)

		intra-assay				inter-assay				CV attributed to final result
			
		Mean Ct	Copy number estimate			Mean Ct	Copy number estimate			
					
	Amount of DNA		Mean	SD	CV		Mean	SD	CV	
lectin	100 ng (87000 copies)	22.12	76459	2652.86	3.47	22.21	76665	2136.80	2.79	7.3
	20 ng (17400 copies)	24.26	19179	514.26	2.68	24.26	19561	562.87	2.88	4.4
	5 ng (4350 copies)	26.38	4861	415.49	8.55	26.39	4705	145.13	3.08	12.5
	1 ng (870 copies)	28.98	895	32.08	3.58	28.89	884	38.35	4.34	10.4
	0.5 ng (435 copies)	30.25	392	20.92	5.33	30.06	403	13.71	3.40	10.9

RRS	100 ng (4350 copies)	27.99	4349	87.08	2.00	28.00	4017	273.05	6.80	
	20 ng (870 copies)	30.46	827	70.50	8.52	30.34	893	29.61	3.31	
	5 ng (218 copies)	32.39	224	23.36	10.41	32.51	223	27.00	12.10	
	1 ng (44 copies)	34.73	47	8.48	18.13	34.92	49	4.69	9.49	
	0.5 ng (22 copies)	35.90*	22	5.84	27.17	36.18*	22	2.30	10.34	

**Table 2:** PCR efficiency for different soybean and maize matrixes. PCR efficiencies are listed for different soybean and sample matrixes for the species specific gene and the transgene (CV = coefficient of variation [%]).

		SPECIES SPECIFIC GENE			TRANSGENE		
	Sample	slope of the standard curve	PCR efficiency	correlation coefficient (R^2^)	slope of the standard curve	PCR efficiency	correlation coefficient (R^2^)

SOYBEAN	CRM	-3.51	0.93	0.9983	-3.46	0.95	0.9926
	grain	-3.83	0.82	0.9982	-3.56	0.91	0.9528
	feed	-3.43	0.96	0.9933	-3.15	1.08	0.9520
	soybean milk	-3.46	0.95	0.9946	-3.69	0.87	0.9664

	CV		7.05	0.25		9.56	1.96

MAIZE	CRM	-3.45	0.95	0.9989	-3.23	1.04	0.9878
	grain	-3.26	1.03	0.9960	-3.10	1.10	0.9570
	feed	-3.19	1.06	0.9794	-3.23	1.04	0.8736
	tortilla chips	-3.31	1.01	0.9982	-3.40	0.97	0.9743

	CV		4.59	0.93		5.12	5.41

**Table 3:** Effect of differences in PCR efficiency on the estimation of GMO content. Effect of changes in real-time PCR efficiency in the sample on the determination of GMO content is shown. The PCR efficiency of the standard curve for both amplicons was set at 1. A 5% GM soybean sample was assumed, using approximately 100 ng of sample corresponding to 87000 copies of soybean genome and 4350 copies of transgene. (E = efficiency, Ct = Cycle threshold)

SPECIES SPECIFIC GENE				TRANSGENE				GMO % ESTIMATE	
E(amplicon)	Slope	expected Ct	Estimation of the initial copy number	E(amplicon)	Slope	expected Ct	Estimation of the initial copy number	% of GMO	% of GMO, varying eff. of species specific gene only

1.15	3.01	19.92	367470	1.15	3.01	23.84	24384	6.64	1.18
1.1	3.10	20.55	237153	1.1	3.10	24.59	14440	6.09	1.83
1.05	3.21	21.24	147004	1.05	3.21	25.42	8148	5.54	2.96
1	3.32	22.00	87000	1	3.32	26.32	4350	5.00	5.00
0.95	3.45	22.83	48803	0.95	3.45	27.32	2178	4.46	8.91
0.9	3.59	23.76	25720	0.9	3.59	28.43	1012	3.94	16.91
0.85	3.74	24.79	12596	0.85	3.74	29.66	431	3.42	34.53

* The % of GMO was calculated assuming a PCR efficiency for the transgene amplicon equal to that for the standard curve (= 1.0); only the efficiency of the species specific gene was assumed to change.

**Table 4:** Primers and fluorogenic probes for Real-time PCR

Target/amplicon length	Specificity/sequence accession number	Primer and probe names	Primer/probe sequence	Lengh (bp)	Reference
Lectin 81 bp	soybean specific gene (Accession number K00821)	TM-Lectin-F	5'-tcc acc ccc atc cac att t-3'	19	29
		TM-Lectin-R	5'-ggc ata gaa ggt gaa gtt gaa gga-3'	24	
		Lectin-FAM	5'-FAM-aac cgg tag cgt tgc cag ctt cg-TAMRA-3'	23	

Invertase 79 bp	maize specific gene (Accession number U16123)	ivr1-TM1	5'-tgg cgg acg acg act tgt-3'	18	30
		ivr1-TM2	5'-aaa gtt tgg agg ctg ccg t-3'	19	
		ivr	5'-VIC-cga gca gac cgc cgt gta ctt cta cc-TAMRA-3'	26	

p35S 82 bp	p35S promoter (Accession number V00141)	TM-35S-1	5'-gcc tct gcc gac agt ggt-3'	18	29, 31
		TM-35S-2	5'-aag acg tgg ttg gaa cgt ctt c-3'	22	
		35S	5'-FAM-caa aga tgg acc ccc acc cac g-TAMRA-3'	22	

tNOS 151 bp	tNOS terminator (Accession number V00087)	tNOSF	5'-gtc ttg cga tga tta tca tat aat ttc tg-3'	29	32
		tNOSR	5'-cgc tat att ttg ttt tct atc gcg t-3'	25	
			5'-FAM aga tgg gtt ttt atg att aga gtc ccg caa-TAMRA-		
		tNOSpro	3'	30	

GTS 40-3-2 soybean 83 bp	construct specific (Accession number AB209952)	RRS-F	5'-gcc atg ttg tta att tgt gcc at-3'	23	29
		RRS-R	5'-gaa gtt cat ttc att tgg aga gga c-3'	25	
			5'-FAM-ctt gaa aga tct gct aga gtc agc ttg tca gcg-		
		RRS	TAMRA-3'	33	

MON810 maize 115 bp	event specific (Accession number AF434709)	Mon810F1311	5'-cct tca taa cct tcg ccc g-3'	19	33
		Mon810R1311	5'-aat aaa gtg aca gat agc tgg gca-3'	24	
			5'-FAM-acg aag gac tct aac gtt taa cat cct ttg cca-		
		Mon810pro1311	TAMRA-3'	33	

## References

[B1] James C (2005). Highlights of ISAAA briefs no. 34-2005 Global status of commercialized biotech/GM crops. ISAAA.

[B2] Miraglia M, Berdal KG, Brera C, Corbisier P, Holst-Jensen A, Kok EJ, Marvin HJ, Schimmel H, Rentsch J, van Rie JP, Zagon J (2004). Detection and traceability of genetically modified organisms in the food production chain. Food Chem Toxicol.

[B3] Holst-Jensen A, Ronning SB, Lovseth A, Berdal KG (2003). PCR technology for screening and quantification of genetically modified organisms (GMOs). Anal Bioanal Chem.

[B4] Lipp M, Shillito R, Giroux R, Spiegelhalter F, Charlton S, Pinero D, Song P (2005). Polymerase chain reaction technology as analytical tool in agricultural biotechnology. J AOAC Int.

[B5] Bubner B, Gase G, Baldwin IT (2004). Two-fold differences are the detection limit for determining transgene copy numbers in plants by real-time PCR. BMC Biotechnol.

[B6] Brodmann P (2002). DNA-Extraction: Important Step of Quantitative Detection of GMO in Food Matrixes. New Food.

[B7] Holden MJ, Blasic JR, Bussjaeger L, Kao C, Shokere LA, Kendall DC, Freese L, Jenkins GR (2003). Evaluation of extraction methodologies for corn kernel (Zea mays) DNA for detection of trace amounts of biotechnology-derived DNA. J Agric Food Chem.

[B8] Peano C, Samson MC, Palmieri L, Gulli M, Marmiroli N (2004). Qualitative and quantitative evaluation of the genomic DNA extracted from GMO and non-GMO foodstuffs with four different extraction methods. J Agric Food Chem.

[B9] Holst-Jensen A, Berdal KG (2004). The modular analytical procedure and validation approach and the units of measurement for genetically modified materials in foods and feeds. J AOAC Int.

[B10] Hellebrand M, Nagy M, Mörsel JT (1998). Determination of DNA traces in rapeseed oil. Z Lebensm Unters Forsch.

[B11] Jerman S, Podgornik A, Cankar K, Cadez N, Skrt M, Zel J, Raspor P (2005). Detection of processed genetically modified food using CIM monolithic columns for DNA isolation. J Chromatogr A.

[B12] Pauli U, Liniger M, Zimmermann A (1998). Detection of DNA in soybean oil. Z Lebensm Unters Forsch.

[B13] Kay S, Van den Eede G (2001). The limits of GMO detection. Nat Biotechnol.

[B14] Hubner P, Waiblinger HU, Pietsch K, Brodmann P (2001). Validation of PCR methods for quantification of genetically modified plants in food. J AOAC Int.

[B15] Wilson IG (1997). Inhibition and facilitation of nucleic acid amplification. Appl Environ Microbiol.

[B16] Rossen L, Norskov P, Holmstrom K, Rasmussen OF (1992). Inhibition of PCR by components of food samples, microbial diagnostic assays and DNA-extraction solutions. Int J Food Microbiol.

[B17] Peist R, Honsel D, Twieling G, Löffert D (2001). PCR inhibitors in plant DNA preparations. Qiagen news.

[B18] Kubista M, Andrade JM, Bengtsson M, Forootan A, Jonák J, Lind K, Sindelka R, Sjöback R, Sjögreen B, Strömbom L, Ståhlberg A, Zoric N (2006). The real-time polymerase chain reaction. Molec Aspects Med.

[B19] Applied Biosystems (2001). User Bulletin #2: Relative Quantification of Gene Expression. Applied Biosystems.

[B20] Holst-Jensen A, De Loose M, Van den Eede G (2006). Coherence between legal requirements and approaches for detection of genetically modified organisms (GMOs) and their derived products. J Agric Food Chem.

[B21] (2005). ISO 21570: Foodstuffs – Methods of analysis for the detection of genetically modified organisms and derived products – quantitative nucleic acid based methods.

[B22] Burns MJ, Nixon GJ, Foy CA, Harris N (2005). Standardization of data from real-time quantitative PCR methods – evaluation of outliers and comparison of calibration curves. BMC Biotechnol.

[B23] Tichopad A, Dilger M, Schwarz G, Pfaffl MW (2003). Standardized determination of real-time PCR efficiency from a single reaction set-up. Nucleic Acids Res.

[B24] Rutledge RG (2004). Sigmoidal curve-fitting redefines quantitative real-time PCR with the prospective of developing automated high-throughput applications. Nucleic Acids Res.

[B25] Van LT, Paquet N, Calvo E, Cumps J (2005). Improved real-time RT-PCR method for high-throughput measurements using second derivative calculation and double correction. Biotechniques.

[B26] Spoth B, Strauss E (1999). Screening for genetically modified organisms in Food using Promega's Wizard resin. Promega Notes Mag.

[B27] Somma M, Querci M, Jermini M, Van den Eede, Guy (2004). Extraction and purification of DNA. The analysis of food samples for the presence of genetically, modified organisms.

[B28] Bundesamt für Gesundheit, (Eds) (2001). Schweizerisches Lebensmittelbuch. CD-ROM, 311510, BBL-EDMZ 3003, Bern, Switzerland.

[B29] Brodmann PD, Ilg EC, Berthoud H, Herrmann A (2002). Real-time quantitative polymerase chain reaction methods for four genetically modified maize varieties and maize DNA content in food. J AOAC Int.

[B30] Pauli U, Schouwey B, Hubner P, Brodmann P, Eugster A (2001). Quantitative detection of genetically modified soybean and Maize: method evaluation in a Swiss ring trial. Mitt Lebensm Hyg.

[B31] Kuribara H, Shindo Y, Matsouka T, Takubo K, Futo S, Aoki N, Hirao T, Akiyama H, Goda Y, Toyoda M, Hino A (2002). Novel reference materials for quantification of genetically modified maize and soybean. J AOAC Int.

[B32] Holck A, Vaitilingom M, Didierjean L, Rudi K (2002). 5'nuclease PCR for quantitative event specific detection of the genetically modified MON810 MaisGardTM maize. Eur Food Res Technol.

[B33] (2002). AbiPrism 7900 HT Sequence detection system and SDS enterprise database, User guide. Applied Biosystems.

[B34] Bennett MD, Leitch IJ (1997). Nuclear DNA Amounts in Angiosperms – 583 New Estimates. Ann Bot.

[B35] Burns MJ, Valdivia H, Harris N (2004). Analysis and interpretation of data from real-time PCR trace detection methods using quantification of GM soya as a model system. Anal Bioanal Chem.

[B36] Rasmussen R, Meuer S, Wittwer C, Nakagawara K (2001). Quantification on the LightCycler instrument. Rapid Cycle Real-time PCR: Methods and Applications.

[B37] Pfaffl MW (2001). A new mathematical model for relative quantification in real-time RT-PCR. Nucleic Acids Res.

[B38] R Development Core Team (2005). R: A Language and Environment for Statistical Computing (manual). R Foundation for Statistical Computing, Vienna, Austria.

